# Tris(η^5^-cyclo­penta­dien­yl)hafnium(III)

**DOI:** 10.1107/S1600536811014516

**Published:** 2011-04-22

**Authors:** Vladimir V. Burlakov, Perdita Arndt, Anke Spannenberg, Uwe Rosenthal

**Affiliations:** a A. N. Nesmeyanov Institute of Organoelement Compounds, Russian Academy of Sciences, Vavilov Street 28, 119991 Moscow, Russian Federation; b Leibniz-Institut für Katalyse e.V. an der Universität Rostock, Albert-Einstein-Strasse 29a, 18059 Rostock, Germany

## Abstract

In the crystal structure of the title compound, [Hf(C_5_H_5_)_3_], three cyclo­penta­dienyl ligands surround the Hf^III^ atom in a trigonal–planar geometry. The mol­ecule lies on a sixfold inversion axis.

## Related literature

Isotypic (η^5^-C_5_H_5_)_3_Zr was described by Lukens & Andersen (1995 ▶). For (η^5^-C_5_H_5_)_3_ *M*, *M* = Y: see Adam *et al.* (1991 ▶); *M* = Nd: see Eggers *et al.* (1992*a* ▶); *M* = Sm: see Wong *et al.* (1969 ▶), Bel’skii *et al.* (1991 ▶), Eggers *et al.* (1992*b* ▶); *M* = Er, Tm: see Eggers *et al.* (1986 ▶); *M* = Yb: see Eggers *et al.* (1987 ▶); *M* = Ce, Dy, Ho: see Baisch *et al.* (2006 ▶). Unit-cell dimensions of (η^5^-C_5_H_5_)_3_ *M* (*M* = Pr, Pm, Sm, Gd, Tb, Tm, Cm, Bk, Cf) were determined by Laubereau & Burns (1970*a* ▶,*b* ▶).
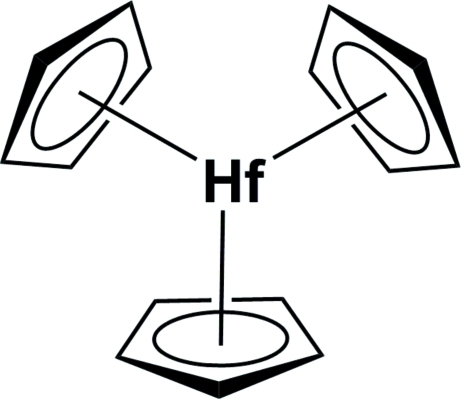


## Experimental

### 

#### Crystal data

[Hf(C_5_H_5_)_3_]
*M* *_r_* = 373.76
Hexagonal, 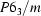

*a* = 7.9772 (4) Å
*c* = 10.2975 (6) Å
*V* = 567.50 (5) Å^3^
*Z* = 2
Mo *K*α radiation
μ = 9.16 mm^−1^
*T* = 150 K
0.30 × 0.20 × 0.15 mm

#### Data collection

Stoe IPDS II diffractometer
Absorption correction: numerical (*X-SHAPE* and *X-RED32*; Stoe & Cie, 2005 ▶) *T*_min_ = 0.150, *T*_max_ = 0.346
7314 measured reflections
362 independent reflections
333 reflections with *I* > 2σ(*I*)
*R*_int_ = 0.097

#### Refinement

*R*[*F* ^2^ > 2σ(*F* ^2^)] = 0.034
*wR*(*F* ^2^) = 0.076
*S* = 1.22
362 reflections
27 parameters
H-atom parameters constrained
Δρ_max_ = 0.95 e Å^−3^
Δρ_min_ = −3.40 e Å^−3^

### 

Data collection: *X-AREA* (Stoe & Cie, 2005 ▶); cell refinement: *X-AREA*; data reduction: *X-AREA*; program(s) used to solve structure: *SHELXS97* (Sheldrick, 2008 ▶); program(s) used to refine structure: *SHELXL97* (Sheldrick, 2008 ▶); molecular graphics: *XP* in *SHELXTL* (Sheldrick, 2008 ▶); software used to prepare material for publication: *SHELXL97*.

## Supplementary Material

Crystal structure: contains datablocks I, global. DOI: 10.1107/S1600536811014516/ng5148sup1.cif

Structure factors: contains datablocks I. DOI: 10.1107/S1600536811014516/ng5148Isup2.hkl

Additional supplementary materials:  crystallographic information; 3D view; checkCIF report

## Figures and Tables

**Fig. 1.**
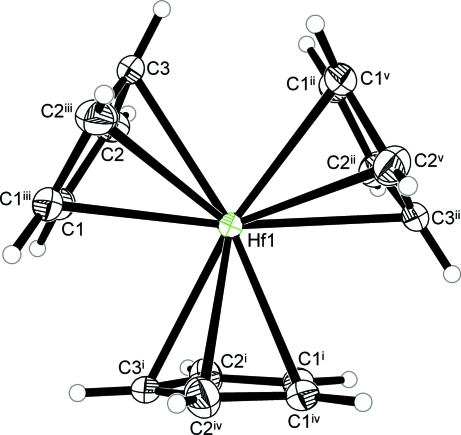
The molecular structure of the title compound showing the atom-labelling scheme. Displacement ellipsoids are drawn at the 50% probability level.

## supplementary crystallographic information

### Comment

In the reaction of (η^5^-C_5_H_5_)_2_Hf[—C(SiMe_3_)═C(C≡CSiMe_3_)— C(SiMe_3_)═C(C≡CSiMe_3_)—] with (*i-*Bu)_2_AlH single crystals of the title compound as lone product in very low yield were isolated. Isostructural compounds are known for *M* = Zr (Lukens *et al.*, 1995), *M* = Y (Adam *et al.*, 1991), *M* = Nd (Eggers *et al.*, 1992*a*), *M* = Sm (Wong *et al.*, 1969; Bel'skii *et al.*, 1991; Eggers *et al.*, 1992*b*), *M* = Er, Tm (Eggers *et al.*, 1986), *M* = Yb (Eggers *et al.*, 1987), *M* = Ce, Dy, Ho (Baisch *et al.*, 2006). (η^5^-C_5_H_5_)_3_Hf crystallizes in the hexagonal space group *P*6_3_/*m* with unit-cell dimensions isomorphous with the Zr analogue (Lukens *et al.*, 1995). The Hf(III) center is surrounded by three η^5^-coordinated cyclopentadienyl ligands in a trigonal planar geometry. The Hf—C distances are with 2.547 (6) and 2.575 (6) Å in the expected range.

### Experimental

An amount of 0.460 g (0.66 mmol) of the five membered metallacycle (η^5^-C_5_H_5_)_2_Hf[—C(SiMe_3_)═C(C≡CSiMe_3_)—C(SiMe_3_)═ C(C≡CSiMe_3_)—] was dissolved in 20 ml of *n*-hexane under Ar, and 2.6 ml (2.6 mmol) of a 1.0 *M* solution of (*i-*Bu)_2_AlH in cyclohexane was added to the obtained yellow solution. After one day the obtained red-brown solution was filtered and allowed to stand in argon atmosphere at -40 °C. After 6 month the light-yellow crystals had formed which were separated from the mother liquor by decanting, washed with cooled *n*-hexane, and dried in vacuum to give (η^5^-C_5_H_5_)_3_Hf. Yield 9.3% (23 mg). *M*.p. 261–263 °C (dec. under Ar). MS (70 eV, m/*z*): 375 (*M*^+^), 310 (*M^+^*-C_5_H_5_).

### Refinement

H atoms were placed in idealized positions with d(C—H) = 0.95 Å and refined using a riding model with *U*_iso_(H) fixed at 1.2 *U*_eq_(C).

A numerical absorption correction was performed. Hence the largest peak of 0.95 (1.57 Å from Hf1) and the deepest hole of -3.40 e Å^-3^ (0.98 Å from Hf1) in the final difference Fourier map were obtained.

### Crystal data

**Table:**

[Hf(C_5_H_5_)_3_]	*D*_x_ = 2.187 Mg m^−^^3^
*M**_r_* = 373.76	Mo *K*α radiation, λ = 0.71073 Å
Hexagonal, *P*6_3_/*m*	Cell parameters from 4609 reflections
Hall symbol: -P 6c	θ = 1.9–28.4°
*a* = 7.9772 (4) Å	µ = 9.16 mm^−^^1^
*c* = 10.2975 (6) Å	*T* = 150 K
*V* = 567.50 (5) Å^3^	Prism, yellow
*Z* = 2	0.30 × 0.20 × 0.15 mm
*F*(000) = 354	

### Data collection

**Table:**

Stoe IPDS II diffractometer	362 independent reflections
Radiation source: fine-focus sealed tube	333 reflections with *I* > 2σ(*I*)
graphite	*R*_int_ = 0.097
ω scans	θ_max_ = 25.0°, θ_min_ = 3.0°
Absorption correction: numerical (*X-SHAPE* and *X-RED32*; Stoe & Cie, 2005)	*h* = −9→9
*T*_min_ = 0.150, *T*_max_ = 0.346	*k* = −9→9
7314 measured reflections	*l* = −12→12

### Refinement

**Table:**

Refinement on *F*^2^	Primary atom site location: structure-invariant direct methods
Least-squares matrix: full	Secondary atom site location: difference Fourier map
*R*[*F*^2^ > 2σ(*F*^2^)] = 0.034	Hydrogen site location: inferred from neighbouring sites
*wR*(*F*^2^) = 0.076	H-atom parameters constrained
*S* = 1.22	*w* = 1/[σ^2^(*F*_o_^2^) + (0.0163*P*)^2^ + 5.6136*P*] where *P* = (*F*_o_^2^ + 2*F*_c_^2^)/3
362 reflections	(Δ/σ)_max_ < 0.001
27 parameters	Δρ_max_ = 0.95 e Å^−^^3^
0 restraints	Δρ_min_ = −3.40 e Å^−^^3^

### Special details

**Table:**

Geometry. All e.s.d.'s (except the e.s.d. in the dihedral angle between two l.s. planes) are estimated using the full covariance matrix. The cell e.s.d.'s are taken into account individually in the estimation of e.s.d.'s in distances, angles and torsion angles; correlations between e.s.d.'s in cell parameters are only used when they are defined by crystal symmetry. An approximate (isotropic) treatment of cell e.s.d.'s is used for estimating e.s.d.'s involving l.s. planes.
Refinement. Refinement of *F*^2^ against ALL reflections. The weighted *R*-factor *wR* and goodness of fit *S* are based on *F*^2^, conventional *R*-factors *R* are based on *F*, with *F* set to zero for negative *F*^2^. The threshold expression of *F*^2^ > σ(*F*^2^) is used only for calculating *R*-factors(gt) *etc*. and is not relevant to the choice of reflections for refinement. *R*-factors based on *F*^2^ are statistically about twice as large as those based on *F*, and *R*- factors based on ALL data will be even larger.

### Fractional atomic coordinates and isotropic or equivalent isotropic displacement parameters (Å^2^)

**Table:**

	*x*	*y*	*z*	*U*_iso_*/*U*_eq_	
Hf1	0.3333	0.6667	0.2500	0.0342 (3)	
C1	0.4331 (9)	0.4179 (9)	0.1824 (6)	0.0236 (13)	
H1	0.5434	0.4592	0.1283	0.028*	
C2	0.2408 (10)	0.3460 (10)	0.1393 (7)	0.0300 (15)	
H2	0.1992	0.3347	0.0517	0.036*	
C3	0.1229 (14)	0.2944 (13)	0.2500	0.026 (2)	
H3	−0.0143	0.2344	0.2500	0.032*	

### Atomic displacement parameters (Å^2^)

**Table:**

	*U*^11^	*U*^22^	*U*^33^	*U*^12^	*U*^13^	*U*^23^
Hf1	0.0126 (3)	0.0126 (3)	0.0772 (6)	0.00632 (15)	0.000	0.000
C1	0.022 (3)	0.019 (3)	0.031 (3)	0.011 (3)	0.003 (3)	−0.002 (3)
C2	0.024 (3)	0.027 (4)	0.035 (4)	0.010 (3)	−0.004 (3)	−0.001 (3)
C3	0.017 (4)	0.014 (4)	0.048 (6)	0.008 (4)	0.000	0.000

### Geometric parameters (Å, °)

**Table:**

Hf1—C2^i^	2.549 (7)	Hf1—C1	2.576 (6)
Hf1—C2^ii^	2.549 (7)	Hf1—C1^ii^	2.576 (6)
Hf1—C2^iii^	2.549 (7)	C1—C1^ii^	1.392 (12)
Hf1—C2^iv^	2.549 (7)	C1—C2	1.414 (9)
Hf1—C2	2.549 (7)	C1—H1	0.9500
Hf1—C2^v^	2.549 (7)	C2—C3	1.402 (9)
Hf1—C1^i^	2.576 (6)	C2—H2	0.9500
Hf1—C1^iii^	2.576 (6)	C3—C2^ii^	1.402 (9)
Hf1—C1^iv^	2.576 (6)	C3—H3	0.9500
Hf1—C1^v^	2.576 (6)		
			
C2^i^—Hf1—C2^ii^	101.55 (19)	C1^i^—Hf1—C1^v^	122.45 (4)
C2^i^—Hf1—C2^iii^	53.1 (3)	C1^iii^—Hf1—C1^v^	112.98 (12)
C2^ii^—Hf1—C2^iii^	126.86 (8)	C1^iv^—Hf1—C1^v^	31.4 (3)
C2^i^—Hf1—C2^iv^	101.55 (19)	C2^i^—Hf1—C1	152.1 (2)
C2^ii^—Hf1—C2^iv^	101.55 (19)	C2^ii^—Hf1—C1	52.7 (2)
C2^iii^—Hf1—C2^iv^	126.86 (8)	C2^iii^—Hf1—C1	130.0 (2)
C2^i^—Hf1—C2	126.86 (8)	C2^iv^—Hf1—C1	94.8 (2)
C2^ii^—Hf1—C2	53.1 (3)	C2—Hf1—C1	32.0 (2)
C2^iii^—Hf1—C2	101.55 (19)	C2^v^—Hf1—C1	81.0 (2)
C2^iv^—Hf1—C2	126.86 (8)	C1^i^—Hf1—C1	122.45 (4)
C2^i^—Hf1—C2^v^	126.86 (8)	C1^iii^—Hf1—C1	112.98 (12)
C2^ii^—Hf1—C2^v^	126.86 (8)	C1^iv^—Hf1—C1	122.45 (4)
C2^iii^—Hf1—C2^v^	101.55 (19)	C1^v^—Hf1—C1	112.98 (12)
C2^iv^—Hf1—C2^v^	53.1 (3)	C2^i^—Hf1—C1^ii^	130.0 (2)
C2—Hf1—C2^v^	101.55 (19)	C2^ii^—Hf1—C1^ii^	32.0 (2)
C2^i^—Hf1—C1^i^	32.0 (2)	C2^iii^—Hf1—C1^ii^	152.1 (2)
C2^ii^—Hf1—C1^i^	81.0 (2)	C2^iv^—Hf1—C1^ii^	81.0 (2)
C2^iii^—Hf1—C1^i^	52.7 (2)	C2—Hf1—C1^ii^	52.7 (2)
C2^iv^—Hf1—C1^i^	130.0 (2)	C2^v^—Hf1—C1^ii^	94.8 (2)
C2—Hf1—C1^i^	94.8 (2)	C1^i^—Hf1—C1^ii^	112.98 (12)
C2^v^—Hf1—C1^i^	152.2 (2)	C1^iii^—Hf1—C1^ii^	122.45 (4)
C2^i^—Hf1—C1^iii^	52.7 (2)	C1^iv^—Hf1—C1^ii^	112.98 (12)
C2^ii^—Hf1—C1^iii^	94.8 (2)	C1^v^—Hf1—C1^ii^	122.45 (4)
C2^iii^—Hf1—C1^iii^	32.0 (2)	C1—Hf1—C1^ii^	31.4 (3)
C2^iv^—Hf1—C1^iii^	152.2 (2)	C1^ii^—C1—C2	108.3 (4)
C2—Hf1—C1^iii^	81.0 (2)	C1^ii^—C1—Hf1	74.32 (14)
C2^v^—Hf1—C1^iii^	130.0 (2)	C2—C1—Hf1	72.9 (4)
C1^i^—Hf1—C1^iii^	31.4 (3)	C1^ii^—C1—H1	125.9
C2^i^—Hf1—C1^iv^	81.0 (2)	C2—C1—H1	125.9
C2^ii^—Hf1—C1^iv^	130.0 (2)	Hf1—C1—H1	118.7
C2^iii^—Hf1—C1^iv^	94.8 (2)	C3—C2—C1	107.3 (6)
C2^iv^—Hf1—C1^iv^	32.0 (2)	C3—C2—Hf1	75.3 (5)
C2—Hf1—C1^iv^	152.2 (2)	C1—C2—Hf1	75.0 (4)
C2^v^—Hf1—C1^iv^	52.7 (2)	C3—C2—H2	126.4
C1^i^—Hf1—C1^iv^	112.98 (12)	C1—C2—H2	126.4
C1^iii^—Hf1—C1^iv^	122.45 (4)	Hf1—C2—H2	115.6
C2^i^—Hf1—C1^v^	94.8 (2)	C2—C3—C2^ii^	108.7 (8)
C2^ii^—Hf1—C1^v^	152.2 (2)	C2—C3—Hf1	73.0 (5)
C2^iii^—Hf1—C1^v^	81.0 (2)	C2^ii^—C3—Hf1	73.0 (5)
C2^iv^—Hf1—C1^v^	52.7 (2)	C2—C3—H3	125.6
C2—Hf1—C1^v^	130.0 (2)	C2^ii^—C3—H3	125.6
C2^v^—Hf1—C1^v^	32.0 (2)	Hf1—C3—H3	120.2

Symmetry codes: (i) −*x*+*y*, −*x*+1, −*z*+1/2; (ii) *x*, *y*, −*z*+1/2; (iii) −*x*+*y*, −*x*+1, *z*; (iv) −*y*+1, *x*−*y*+1, −*z*+1/2; (v) −*y*+1, *x*−*y*+1, *z*.
